# *De Novo* Assembly of Human Herpes Virus Type 1 (HHV-1) Genome, Mining of Non-Canonical Structures and Detection of Novel Drug-Resistance Mutations Using Short- and Long-Read Next Generation Sequencing Technologies

**DOI:** 10.1371/journal.pone.0157600

**Published:** 2016-06-16

**Authors:** Timokratis Karamitros, Ian Harrison, Renata Piorkowska, Aris Katzourakis, Gkikas Magiorkinis, Jean Lutamyo Mbisa

**Affiliations:** 1 Department of Zoology, University of Oxford, Oxford, United Kingdom; 2 Virus Reference Department, National Infection Services, Public Health England, London, United Kingdom; CNRS UMR7622 & University Paris 6 Pierre-et-Marie-Curie, FRANCE

## Abstract

Human herpesvirus type 1 (HHV-1) has a large double-stranded DNA genome of approximately 152 kbp that is structurally complex and GC-rich. This makes the assembly of HHV-1 whole genomes from short-read sequencing data technically challenging. To improve the assembly of HHV-1 genomes we have employed a hybrid genome assembly protocol using data from two sequencing technologies: the short-read Roche 454 and the long-read Oxford Nanopore MinION sequencers. We sequenced 18 HHV-1 cell culture-isolated clinical specimens collected from immunocompromised patients undergoing antiviral therapy. The susceptibility of the samples to several antivirals was determined by plaque reduction assay. Hybrid genome assembly resulted in a decrease in the number of contigs in 6 out of 7 samples and an increase in N(G)50 and N(G)75 of all 7 samples sequenced by both technologies. The approach also enhanced the detection of non-canonical contigs including a rearrangement between the unique (UL) and repeat (T/IRL) sequence regions of one sample that was not detectable by assembly of 454 reads alone. We detected several known and novel resistance-associated mutations in UL23 and UL30 genes. Genome-wide genetic variability ranged from <1% to 53% of amino acids in each gene exhibiting at least one substitution within the pool of samples. The UL23 gene had one of the highest genetic variabilities at 35.2% in keeping with its role in development of drug resistance. The assembly of accurate, full-length HHV-1 genomes will be useful in determining genetic determinants of drug resistance, virulence, pathogenesis and viral evolution. The numerous, complex repeat regions of the HHV-1 genome currently remain a barrier towards this goal.

## Introduction

Human herpesvirus type 1 (HHV-1), also known as Herpes simplex virus type 1 (HSV-1), has seroprevalence that ranges from 60 to 90% in the general population [1]. Despite the majority of the infections being asymptomatic, 15 to 45% of the adult population suffers from recurrent labial lesions [2]. In addition, encephalitis and corneal keratitis occur in one per 500,000 and in 30 per 100,000 people per year, respectively [3, 4]. The virus is also increasingly being associated with genital lesions [5–7] but no effective vaccine is available at the moment [8].

However, several drugs are licensed for the treatment of recurrent HHV-1 infection in immunocompromised individuals as well as prophylaxis in patients undergoing bone marrow or solid organ transplantation. Antiviral drugs used include the nucleoside analogs acyclovir (ACV)–the drug of choice -, and penciclovir (PCV), as well as foscarnet (FOS), a pyrophosphate analog [9]. The mechanism of these drugs is through inhibition of the viral DNA polymerase (Pol) by acting as competitive inhibitors and/or as chain polymerization terminators. The mono-phosphorylated nucleoside analog Cidofovir (CDV) also inhibits Pol, but is not approved for the treatment of HHV-1 infections [10]. All of them are prone to the selection of resistance mutations within the viral *pol* gene, but ACV and PCV can mainly become ineffective due to the selection of mutations within the thymidine kinase (*TK*) gene, which is essential for their initial activation [11, 12]. Mutations that are selected in TK are normally insertions or deletions in homopolymeric regions (runs of Gs or Cs) that result in frameshift mutations and premature stop codons [13].

The genome of HHV-1 is a long -152kbp-, double-stranded DNA molecule with a high G/C content of 68%. It is unequally divided into one long (L) and one short (S) region. Each region contains a unique sequence, called unique long (UL) and unique short (US), which have only one copy per genome and are both flanked by terminal -T- and internal -I- repeated sequences (TLR/ILR, ISR/TSR). These repeats are characterized by low conservation rates, incorporating numerous microsatellite loci, known as Variable Number Tandem Repeats (VNTRs) and palindrome stem loops (oriL and oriS) omnipresent among the genome. A terminal redundancy of approximately 400 bp, known as *a’* sequence, is located at the ends of this linear genome, but also merges the L and the S segments [14, 15]. The inverted repeats and the *a’* sequence domain play a pivotal role in the recombination events that occur between the L and the S segments [16, 17]. These events are thought to be essential for the viral replication and the *in-vivo* infection [18, 19]. HHV-1 strains vary by geographic region, between individuals but also over sequential isolates from the same individual [20, 21]. The repetitive elements are mainly responsible for this heterogeneity and make the full HHV-1 genome-determination a real challenge, even with the use of high-throughput sequencing technology [22]. The need to address these sequencing limitations is obvious due to the fact that the repetitive elements are also located within coding regions and, in some cases, are well conserved among different strains [22]

To date, several genome sequences of HHV-1, including strain 17 [14, 15], strain KOS [23, 24] and strain McKrae [25, 26], have been described in detail [27–29]. Studies of larger HHV-1 genomes pools (n = 7) [30] have implemented a map-to-reference assembly approach. *De novo* assembling approaches generally fail to construct full-length genomes [22]. A larger set of genomes (n = 20) has been successfully described recently, where reference sequences were used only for the mapping-orientation of the *de novo* generated contigs [27]. To date, only Sanger and short-read NGS technology have been used in these studies, with the former thought to be impractical for a genome on these dimensions and the latter, to have issues regards genome assembly especially with the resolution of repetitive elements [22].

Oxford Nanopore Technologies (ONT) recently released “MinION”, a USB3.0-interfaced sequencer -initially available only to the participants of ONT’s Minion Access Program (MAP)- which is capable of producing hundreds of megabases of data per run, delivering extra-long reads, exceeding 100,000bp in length. These reads, despite their low accuracy which did not exceed 72% for the double-strand sequenced reads (2D) in the first version of the sequencer [31], have been used in combination with other sequencing platforms that deliver shorter reads of higher accuracy, to improve the hybrid *de novo* assembly of genomic regions that are difficult to be resolved [32].

In the present study we employ culture-isolated HHV-1 clinical samples, that were phenotypically characterized for antiviral drug susceptibility, and longer read-lengths derived from both Roche 454 GS Junior and Minion sequencing platforms to prove that MinION is capable of improving the reference-free *de novo* assembly of the viral genome and to pass through repetitive elements that terminate the contigs generated by the 454 platform alone. We also show that the use of longer reads reveals rearrangement phenomena in *de novo* assembled contigs that suggest the existence of non-canonical HHV-1 genomes in our cultures.

## Results

### Experimental design

**Fig 1:**
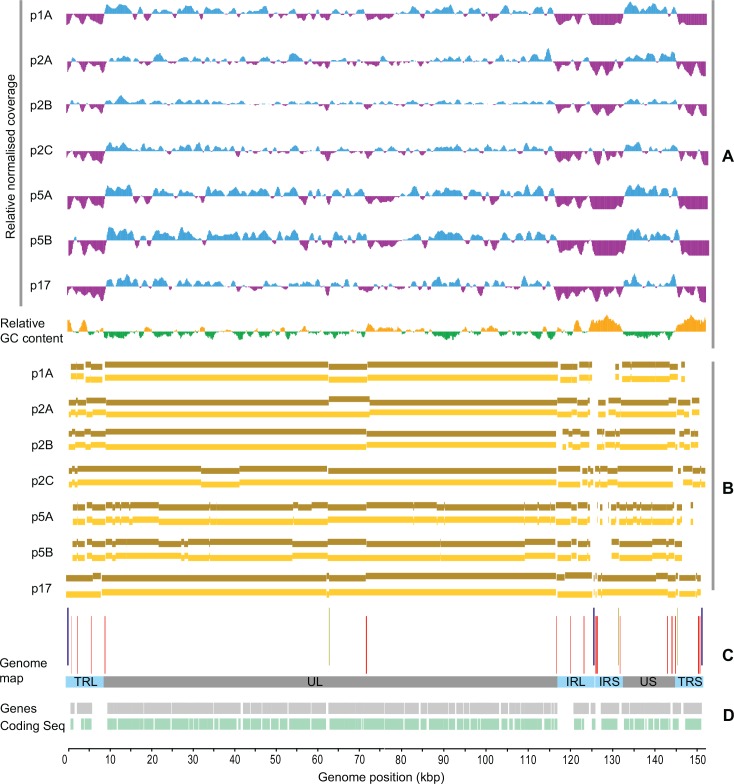
454 read coverage, GC content and comparative mapping of contigs across the genome map. (A): Relative normalized read coverage per sample (454 data) -blue and purple bars represent values beyond and below the average read coverage of the mapping assembly- in correlation with the relative GC content across the reference genome (NC_001806.2) -orange and green bars represent values beyond and below the average GC content- (B): Comparative mapping of solo 454 (brown) and hybrid 454/MinION (yellow) generated contigs. (C): Repetitive elements plotting across the reference genome (NC_001806.2)–“a” sequence in blue, VNTRs in red, stem loops in yellow bars-. (D): Basic structure of the HSV1 genome; UL and US (dark grey) stand for Unique Long and Unique Short region respectively, while TR(L/S) and IR(L/S) (light blue) stand for Terminal and Internal -inverted- Repeats flanging the UL and the US region respectively. Genes (light grey) and coding sequences (light green).

We used two different NGS technologies, these being Roche 454 and MinION, to sequence 18 HHV-1 genomes from cell culture-isolated clinical samples derived from 13 patients. Multiple -sequential- samples were obtained from 3 patients. Seven isolates from 4 patients in total were sequenced by both technologies (Table 1). Each technology required different protocols for library preparation and sequencing. We conceived a hybrid pipeline to filter, combine and evaluate the 454 and the MinION data using MIRA assembler [33] and LINKS [34] as described in the materials and methods section. Using “bedtools”[35] we found that the 454 read coverage across the genome varied. There was a drop in the average normalized coverage observed over genomic regions with higher GC content and vice versa (Fig 1A). This phenomenon was consistent across all samples tested and more pronounced over the terminal and internal repeated sequences (TLR/ILR, ISR/TSR). Most of the discontinuous contigs were also observed over these regions (Fig 1B). For 454 sequencing, the read lengths ranged up to 1,090bp with a mean of 378.96bp ± 45.81. In contrast, for Minion sequencing, the read lengths ranged up to 210,511bp with a mean of 930.62bp ± 526.30 (S1 Fig).

**Table 1:** Solo-454 vs. hybrid 454-MinION *de novo* assembly metrics.

			Statistics without reference					Genome statistics				
	Contigs	Contigs (>1000bp)	Largest contig	Total length	N50	N75	GC (%)	Genome fraction (%)	Duplication ratio	Largest alignment	NG50	NG75
												
p1A_454	21	9	62,373	136,935	45,352	45,352	66.75	89.85	1.099	62,312	45,352	5,665
p1A_454_minion	18	6	111,746	136,978	111,746	111,746	66.65	87.78	1.065	107,664	111,746	6,270
												
p2A_454	26	10	55,661	148,323	45,492	14,325	67.23	95.05	1.126	53,278	45,492	14,325
p2A_454_minion	29	10	108,432	149,533	108,432	13,725	67.28	96.68	1.143	108,421	108,432	13,725
												
p2B_454	39	11	111,943	163,752	111,943	12,873	67.74	95.80	1.204	111,943	111,943	12,873
p2B_454_minion	34	11	111,940	161,088	111,940	15,115	67.56	95.87	1.179	111,940	111,940	15,115
												
p2C_454	22	10	54,727	147,080	30,026	16,316	67.49	92.23	1.087	54,727	30,026	16,316
p2C_454_minion	20	9	76,134	148,065	76,134	17,656	67.53	92.00	1.092	76,133	76,134	17,656
												
p5A_454	66	33	19,559	163,748	5,915	2,005	66.4	91.77	1.272	19,559	7,362	2,339
p5A_454_minion	58	31	20,635	159,894	8,585	2,037	66.59	91.10	1.274	20,635	8,585	2,318
												
p5B_454	39	21	20,715	147,686	8,954	5,706	66.59	90.68	1.181	20,715	8,954	5,577
p5B_454_minion	37	19	26,999	147,043	13,669	5,707	66.58	90.74	1.171	26,999	13,669	5,577
												
p17_454	31	9	62,781	150,948	45,598	13,298	67.7	94.82	1.114	62,778	45,598	13,298
p17_454_minion	29	7	62,781	150,940	45,598	16,151	67.7	91.39	1.111	62,778	45,598	16,151

### The hybrid *de novo* assembly of HHV-1 genomes using 454 and MinION data

**Fig 2:**
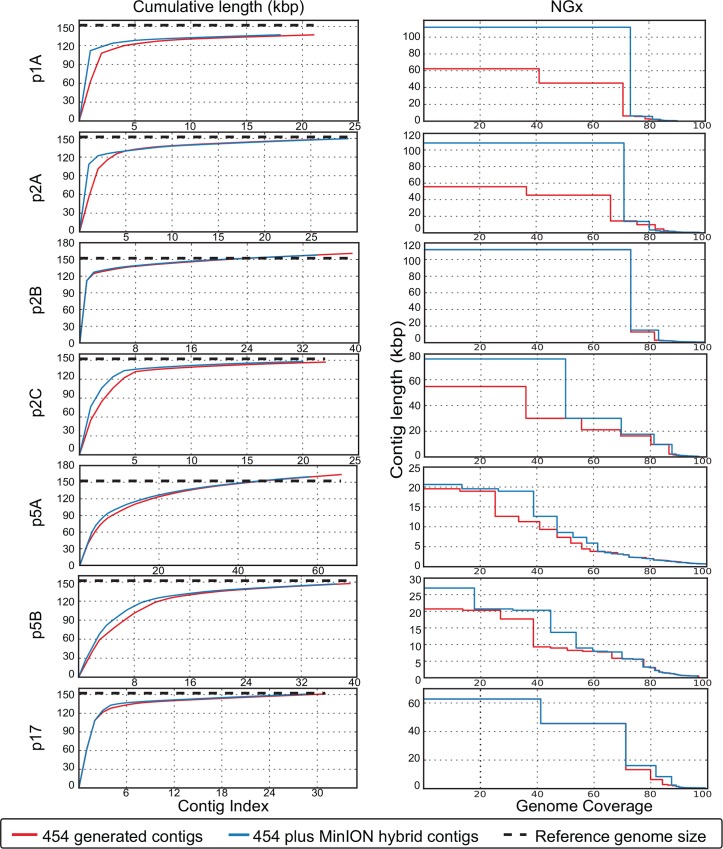
Evaluation of solo 454 and hybrid 454/MinION de-novo assemblies. Contigs Generated by 454 reads are in red while hybrid contigs generated by 454 and MinION reads are in blue. Left column: Cumulative assembly length plotted against the number of the contigs of each individual assembly. Right column: NGx value resulted by the alignment of the contigs on the reference HHV-1 genome.

We found that the *de novo* assembly of the 454-derived HHV-1 genomes was improved by the use of MinION reads through a hybrid assembly protocol. In detail, the number of contigs was reduced while the N(G)50 and the N(G)75 increased across all the 7 samples sequenced by both technologies, with the exception of sample p2A, where the number of the hybrid 454-MinION contigs increased (29 vs. 26 for the 454 contigs). This was probably due to the increased host (VERO cells) contaminating genomic DNA in this particular sample, since only 0.77% of the total MinION reads were mapped on HHV-1 reference (S1 Fig). As the extra contigs were smaller than 1,000bp, they only influenced the N75 value of the hybrid assembly (13,725 vs. 14,325 for the 454 contigs), while two larger contigs were merged, leading to an increased N50 value (108,432 vs. 45,492 for the 454 contigs). No other association between the host genomic DNA and the outcome of the assemblies was observed. The generation of the larger contigs was also assisted by the MinION reads in all but 2 cases, where the larger contig was not improved (samples p2B and p17). The genome coverage remained almost unchanged in most of the samples, while it was reduced by 2.1% and 3.4% in the hybrid assemblies of samples p1A and p17 (Table 1 and Fig 2).

### The effect of hybrid assembly on deciphering structural variability and non-canonical contigs of HHV-1 genomes

**Fig 3:**
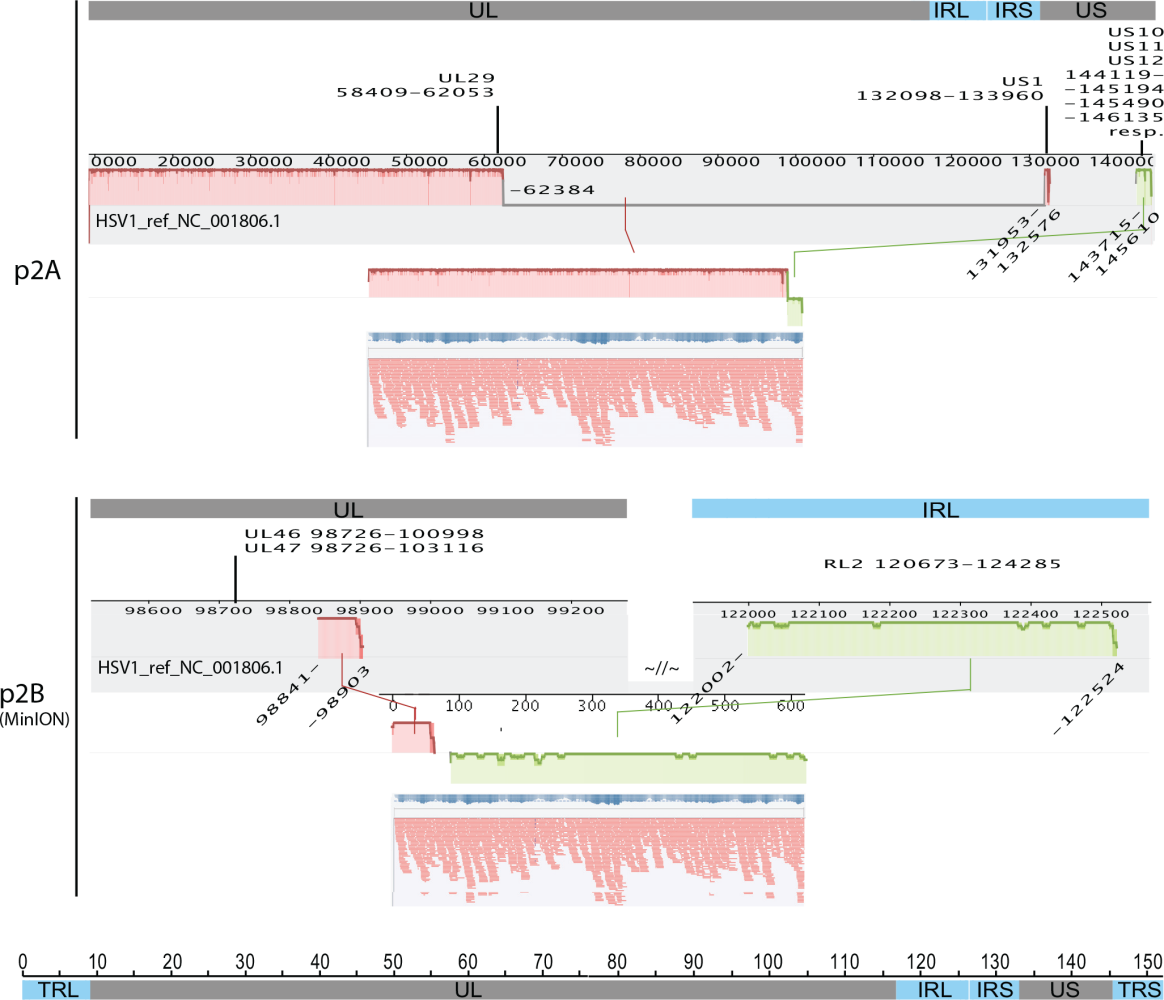
Detection of non-canonical contigs. Similarity plots between 2 representative non-canonical contigs (middle panel of each screenshot) and the reference genome (strain 17) (top panel of each screenshot). Plots below the base line indicate inverted sequences (plus/minus strand alignment). The coordinates of the edges of each rearranged fragment are below while the coordinates of the disrupted/nearby genes are above the reference plots. Confirmative 454-mapping alignments and coverage of the respective supporting reads are shown below each contig.

To investigate if MinION sequencer is capable of enhancing the mining of non-canonical HHV-1 genomes, we used MAUVE [36] to compare misaligned contigs (those that are not aligned in a canonical way to the reference during the assembly evaluation with QUAST [37]) generated from solo-454 and hybrid 454-MinION assemblies. We identified a total of 8 non-canonical contigs in 5 out of 7 samples that were used in the hybrid assemblies. One of these contigs was identified only in the hybrid assembly of sample p2B and not in the solo-454 assembly. The remaining non-canonical contigs were identified in both the solo-454 and the hybrid assemblies. We confirmed these contigs by using them as reference sequences and mapping the 454 reads against them, to visually inspect the continuity of the mapping assembly over the rearrangement junctions. Detailed analysis revealed possible rearrangement events only within the UL region of samples p1A, p17 and in one contig of p2A while the second identified contig of sample p2A and the contigs found in sample p5A suggested rearrangements between the UL and the T/IRS regions. In the case of sample p2B the identified contigs suggested rearrangements between the UL and the T/IRL regions but also between the T/IRL and the T/IRS regions (Fig 3).

### Genetic variability and detection of mutations associated with antiviral resistance across the HHV-1 genome

**Fig 4:**
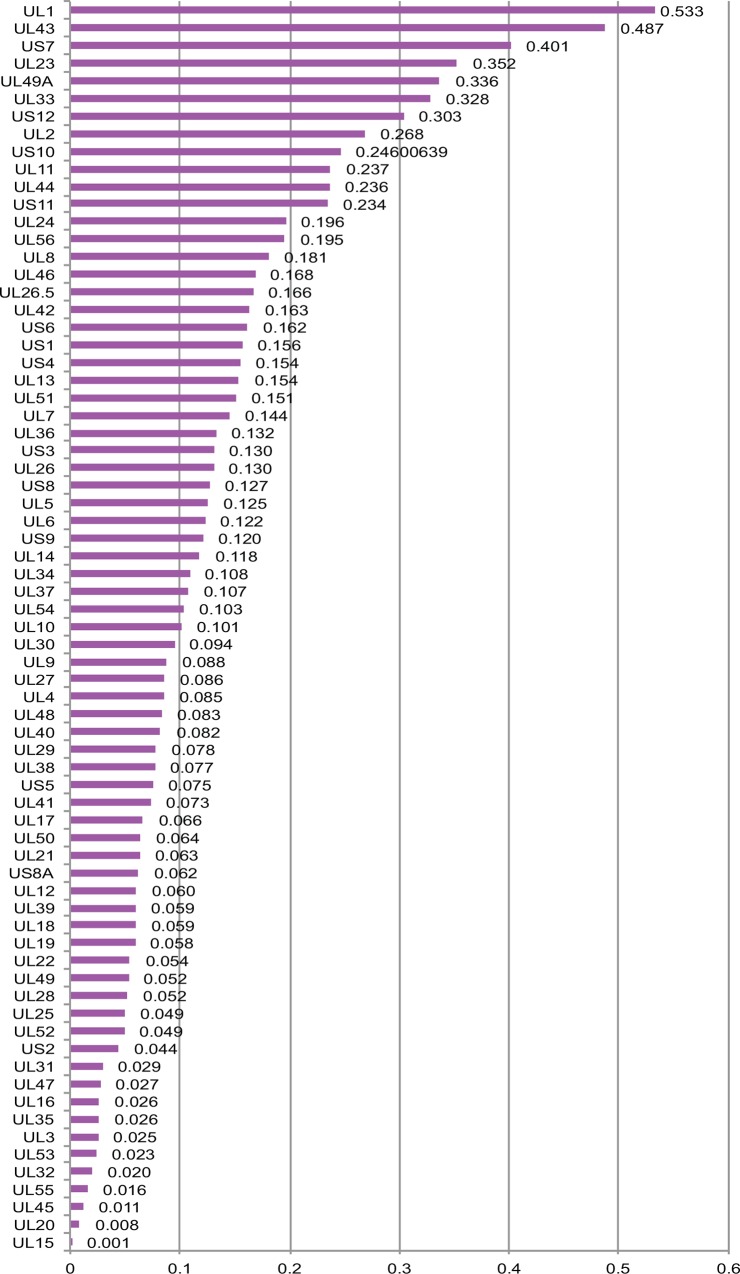
Conservation of the sequenced genomes. Relative -cumulative- amino acid mutation frequency across the HHV-1 reference genes. RL1, RS1 and RS2 have been omitted due to reduced read coverage over the inverted repeated regions

Full genome sequencing of HHV-1 samples will be useful in understanding the full extent of the natural intra- and inter-host sequence variability as well as variability resulting from selective pressure e.g. antiviral therapy. Using *samtools* package [38] snpEff (v3.6c) [39] and snpSift (v3.6c) [40] for variation calling and filtering, and R scripts for basic computations, we estimated the average mutation rate per gene across the HHV-1 genome in these samples taken from patients undergoing antiviral therapy. We omitted the three genes (RL1, RL2, RS1) included in the TLR/ILR and ISR/TSR regions from this analysis due to the lower read coverage over these regions which resulted in fragmented open reading frames. UL15, UL20, UL45, UL55 and UL32 were the 5 most conserved genes as only 0.1%, 0.8%, 1.1%, 1.6% and 2.0% of their amino acids had been mutated at least once within the sample pool, while UL1, UL43, US7, UL23 and UL49A genes accumulated the majority of the non-synonymous mutations across the genome, with 53.3%, 48.7%, 40.1%, 35.2% and 33.6% amino acid changes respectively (Fig 4).

Next, we investigated amino acid changes and genetic variability that could be associated with the development of antiviral resistance. We identified several known and novel mutations in the TK and/or DNA polymerase genes of the samples exhibiting reduced phenotypic antiviral drug susceptibility (Table 2). Previously identified mutations that were detected include A719V and S724N in UL30; A93V, H58R, Q250stop and several single nucleotide insertions resulting in a premature stop codon in UL23 [9, 13, 41–43]. Two novel mutations were identified in UL23, these being V204G and Y172S. The former is located in a non-conserved region of the gene whereas the latter is located within the nucleotide-binding site and C and F substitutions at that position have been previously described to result in ACV resistance [41, 42].

**Table 2:** Phenotypic drug susceptibility testing in HHV-1 clinical samples. Natural polymorphisms and drug resistance mutations in TK and Pol genes.

Sample	Clinical Info	Time	Drug susceptibility profile ^a^				Thymidine Kinase (TK-UL23) ^b^			Polymerase (Pol-UL30) ^b^		
		(Follow up, days)	ACV	PCV	FOS	CDV	Natural Polymorphisms	Drug Resistance Mutations	References for TK DRMs	Natural Polymorphisms	Drug Resistance Mutations	Reference for Pol DRMs
p1A	bone marrow transplant; ACV therapy	0	**>40**	9.67	92.13	2.84	C6G, N23S, K36E, L42P, A265T, W293R, V348I	**A93**	Novel, (Schubert et al., 2014: unclear)	S33G, **E860K**, V905M, **G985E** P1124H, T1208A	None	
p1B		147	1.17	2.86	86.86	15.46	C6G, N23S, K36E, L42P, A265T, W293R, V348I	None		S33G, **E860K,** V905M, **G985E**, P1124H, T1208A	None	
p2A	recurrent HSV keratitis; ACV therapy	0	*21*.*63*	**>160**	<50	6.09	C6G, N23S, K36E, R41H	G ins to 7Gs nt. 430–436, stop codon at AA 224	Andrei et al., 2013	S33G, **E992K,** A1203T, T1208A	None	
p2B		196	1.3	4.05	77.47	13.45	C6G, N23S, K36E, R41H, A192V, G251C, A265T, V267L, P268T, D286E, W293R, N376H	None		S33G, **E992K,** A1203T, T1208A	None	
p2C		535	**>40**	**>160**	162.67	8.75	C6G, N23S, K36E, R41H, A192V, G251C, A265T, V267L, P268T, D286E, N376H	**V204G**	None	S33G, A1203T, T1208A	None	
p2D		780	**>40**	**148.48**	216.31	3.94	C6G, N23S, K36E, R41H, A192V, G251C, A265T, V267L, P268T, D286E, W293R, N376H	**V204G**	None	S33G, **S914L,** A1203T, T1208A,	None	
p3	renal transplant, GVHD; ACV therapy	0	*27*.*37*	**>160**	100	3.61	E146G	A ins at nt. 438, stop codon at AA 227	Andrei et al., 2013	S33G, A562T, D672N, V905M, P1124H, T1208A	None	
p5A	bone marrow transplant; ACV therapy	0	**>40**	**>160**	85.86	4.13	N23S, K36E, R89Q,	G ins to 7Gs nt. 430–436, stop codon at AA 224	Andrei et al., 2013	S33G, D672N, V905M, P920S, T1208A	None	
p5B		8	0.66	4.94	87.83	13.51	N23S, K36E, R89Q, A265T			S33G, D672N, V905M, P920S, T1208A	None	
p6	unknown	0	0.57	2.48	99.28	4.39	A265T, W293R, V348I	None		**S911N,** T1121M, P1124H, T1208A	None	
p7	haematopoietic stem cell transplant; ACV therapy	0	**>40**	**154.4**	97.67	3.47	N23S, K36E, R89Q	G ins to 7Gs nt. 430–436, stop codon at AA 224	Andrei et al., 2013	S33G, T1208A	None	
p9	ACV therapy	0	*35*	**123.48**	86.67	4.46	C6G, N23S, K36E, R41H, A192V, G251C, A265T, V267L, P268T, D286E, W293R, N376H	H58R	Piret & Boivin, 2011	S33G, A429T, V905M, P1124H, T1208A	None	
p10	unknown	0	*34*.*04*	**>160**	95.37	3.5	G240E	Q250stop	Sauerbrei et al., 2011	S33G, A102T, V905M, P920S	None	
p11	post allograft, relapsed acute myeloid leukemia	0	*5*.*91*	5.16	130.43	5.68	C6G, N23S, K36E, R41H, A192V, G251C, A265T, V267L, P268T, D286E, W293R	None		S33G, V905M, A1203T, T1208A,	A719A/V	Piret & Boivin, 2011
p12	eczema herpeticum; ACV therapy	0	**>40**	**>160**	110.82	2.99	G240E, A265T, R281Q, W293R	**Y172S**	None	None	None	
p15	Allogenic haematopoietic stem cell transplant; ACV therapy	0	**>40**	**155.56**	143.24	5.08	C6G, N23S, K36E	G ins to 7Gs nt. 430–436, stop codon at AA 224	Andrei et al., 2013	S33G, V905M, A1203T, T1208A	None	
p17	ACV therapy	0	*5*.*43*	*14*.*6*	**700.78**	**39.58**	C6G, N23S, K36E, L42P, G251C, A265T, W293R	None		S33G, **S239L,** V905M, P1124H, A1204T, T1208A	S724N	Piret & Boivin, 2011
p18	unknown	0	*17*.*08*	*20*.*81*	**681.63**	23.18	G240E, A265T, R281Q	None		S1123L, P1124H, T1208A	S724N	Piret & Boivin, 2011

a: Definitions of phenotypic drug susceptibility classification: Acyclovir (ACV): <3uM, 3-40uM, >40uM; Pencyclovir (PCV): <10uM, 10-40uM, >40uM; Foscarnet (FOS): <250uM, 250-400uM, >400uM; Cidofovir (CDV): <24uM, 24-30uM, >30uM for sensitive (normal type), intermediate (*italics*) and resistant (**bold type**) samples, respectively.

b: Novel drug resistance mutations (DRMs) and novel natural polymorphisms are in bold.

Multiple comparisons among the available sequential samples of patients 1, 2 and 5 did not reveal any difference in their genomic variability, but we were able to identify cooption and also loss of drug resistance mutations during the follow-up (Table 2).

## Discussion

The *de novo* assembly of HHV-1, and other herpesviruses, is challenging due to the increased length and the unique structure of the genome, which incorporates extended regions that are repeated (internal and terminal inverted repeats) and omnipresent VNTRs that usually exceed the read length of existing next generation sequencing platforms [22, 44, 45]. In this study we investigated the potential of a newly released sequencer, the MinION, to improve the *de novo* assembly pipeline of HHV-1 by assisting with the length of contigs generated by the Roche 454 GS Junior Sequencer. We also present relevant information about the structural variability of the samples and we describe how the GC content poses one more barrier towards the full genome sequencing of the HHV-1 genome.

To date, only platforms that deliver shorter read lengths have been used to sequence the HHV-1 genome [22, 46]. The MinION sequencer has been used to successfully improve the *de novo* assemblies of data generated by Illumina HiSeq platforms through the increased length of reads that it produces [32]. Our results suggest for the first time that MinION sequencer is capable of improving the *de novo* assemblies derived from Roche 454 GS Junior sequencer, which delivers increased read lengths (up to ~600bp) compared to Illumina platforms. 454 GS Junior has higher accuracy (reaching 99%) compared to the MinION sequencer (~80%) [32, 47], but the error-profile largely depends on the sequence composition of the template—being less effective over homo-polymeric regions [48], which are quite common across HHV-1 genome.

**Fig 5:**
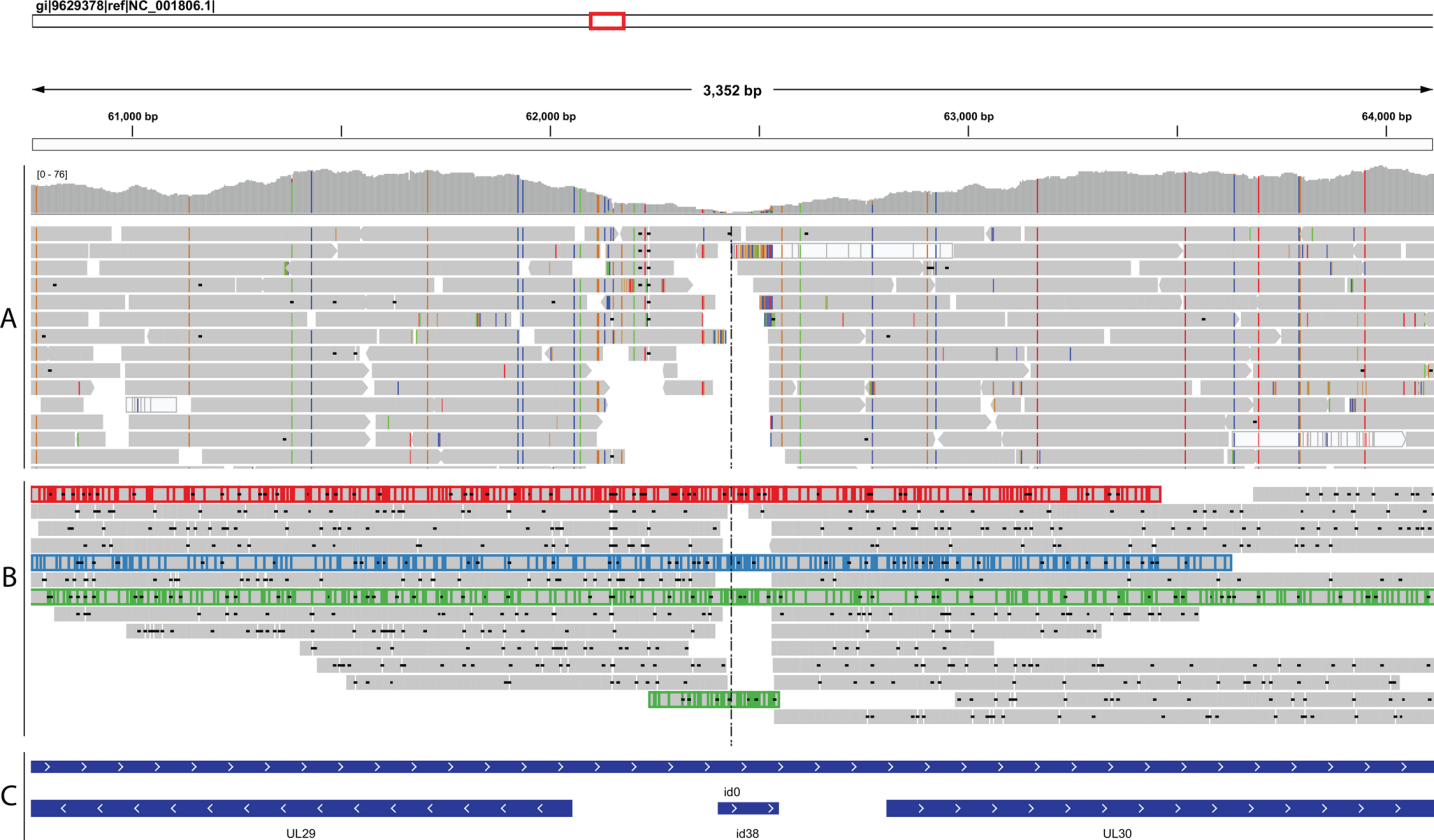
**Mapping of 454-Roche (A) and Nanopore-MinION (B) reads over the oriL palindrome sequence (id38, 62403 – 62547bp in strain 17).** Long MinION reads (in red, blue, green) assist the bridging of discontinuous 454 contigs. (C): genome annotation.

We used MIRA, a whole genome sequence assembler, in all our *de novo* assemblies as it provides the option of hybrid assembly and it has been reported to deliver longer contigs (N50~120,000bp) for Roche 454 GS Junior reads [48]. In most of the samples tested in this study there was an overall improvement in the hybrid assemblies with regards to the N(G)50/75 values and the larger contig generated, compared to the corresponding solo-454 assemblies. The minor drop in the genome coverage observed in some cases, maybe due to multiple mappings of -small- 454 contigs that were merged into larger ones in the hybrid assemblies. This is highly likely due to the repetitive nature of the HHV-1 genome that gives the opportunity for smaller contigs to map into multiple positions. For samples p2A and p2C, we managed to merge the two largest contigs of the 454 assemblies, as the MinION reads transverse the oriL palindrome sequence (62,403–62,547bp in strain 17) (Figs 2 and 5). This region of the HHV1 genome is difficult to read through, as most of the 454-generated assemblies failed to generate a continuous contig through it. A possible reason for this failure is the lower read coverage that was consistently observed over the palindrome (Fig 1), which might be caused by the inability to read sequence over the secondary structure that it forms. Secondary structures are a common problem in Pyrosequencing as they give alternative priming sites on the template and thereby generate high background noise [49]. This is not the case for the MinION sequencer, which has specially developed chemistry for analysing only single-stranded DNA molecules through its nanopores, thus denaturing secondary structures. The extensive length of the MinION reads allowed the merging of contigs that were disrupted by repetitive elements across the HHV-1 genome (Fig 1 and S2 Fig).

Based on the technique of molecular combing, structural variances and non-canonical genomes that incorporate internal duplications, deletions or rearrangements are shown to be present in HHV-1 cultures [50]. Here we identify these events, based on non-canonical contigs that are generated from the reference-free, *de novo* assembly of 454 and MinION reads. One of these contigs was identified only in the hybrid 454/MinION assembly of sample p2B (Fig 3). The reference-free approach allows the mining of minority reads that correspond to the structural variances of the viral genome, which would otherwise have been ignored using the reference sequence. We provide strong evidence of the existence of these structural variances by using the identified non-canonical contigs as reference sequences and by re-mapping the 454 reads against them. This allowed the mapping of 454 reads that had been ignored during the reference mapping assembly of the respective samples. Visual inspection of the original alignments over the coordinates that correspond to the recombination points of the non-canonical contigs, confirmed the continuity of the alignments, suggesting the co-existence of the canonical and non-canonical genomes in the same sample. This observation is also supported by a recent study [27].

The GC content across the HHV-1 genome has been correlated with reduced read coverage regions in mapping assemblies [30]. Most of the times these regions were the inverted IRS and TRS, which also incorporate numerous VNTRs. Our analysis is consistent with the study of Szpara et al [22] which demonstrated that VNTRs were responsible for disrupting the *de novo* assembly of contigs both for strain F and for the clinical isolate H129. The relative normalized read coverage in our mapping assemblies tended to be reduced wherever the relative GC content across the reference genome was increased in all of the 18 454-sequenced samples (Fig 1, only the seven 454- and MinION-sequenced samples presented). MinION reads did not substantially assist the merging of contigs within the demanding inverted repeats, especially the IRS and the TRS. These regions are characterised by low complexity, which in combination with the low accuracy of MinION reads, may have resulted in conflicting assemblies. It is also possible that these regions might by polymorphic among quasispecies within the same sample, which would then produce conflicting assemblies. This will probably be improved by the new MinION sequencer version, which has decreased sequencing error rates and improved sequencing accuracy.

Although nucleotide differences are dispersed throughout the HHV-1 genome, the genes are not equally conserved. The filtered non-synonymous mutations mined from 18 samples in this study, revealed that some genes differ substantially from the reference (cumulative up to 53.3%) while others were extremely conserved (cumulative down to 0.1%). Ushijima et al [28], describing HHV-1 mutant HF10, also showed a relatively high divergence in proteins UL1, UL2, UL11, UL44, US1, US7, US8.5, US10 and US12. Kolb et al [30] sequenced 7 samples in a multiplex format and reported UL43, UL1, UL49A, UL11, US4 and US7 as the most variable genes. Our observations were similar, with the exception of US4 gene, which demonstrated only moderate variation in our samples and UL23 gene, which was the 4^th^ most variable in our samples but had moderate variation in the same study (yet still, within the 10 more variable genes). UL23 (TK gene) is where the majority (>90%) of the drug resistance mutations occur. This might be correlated with the fact that the cohort studied here is of patients undergoing antiviral therapy. The size and the demographic characteristics of the sample pools but also the different informatics pipelines used for the variation calling may explain the differences observed between the two studies. Genes UL16, UL15, UL45, UL28 and UL3, which were reported among the most conserved, also demonstrated low variation levels (up to 5.2%) in our study (Fig 4).

Our results indicate that future relevant studies focusing on the resolution of the VNTRs and the repetitive regions of the genome should make use of longer reads derived from 3^rd^ generation sequencers like MinION. Hopefully, increased accuracy of the long-read technology, in combination with the depth of short-read platforms, will improve the full genome *de novo* assembly of HHV-1, which remains a challenging task.

## Materials and Methods

### Cell culture isolation and phenotypic drug susceptibility testing of HHV-1 clinical samples

In this study, we used a total of 18 HHV-1 isolates from 13 different patients (Table 2). The viruses were isolated from clinical swabs taken from patient lesions by the attending clinician and sent to the National Reference Laboratory at Public Health England for routine diagnosis and characterization of drug resistance. The viruses were isolated and titrated using monolayers of African green monkey kidney cells (Vero cells). Phenotypic antiviral susceptibility testing for HHV-1 was performed as previously described [51]. Briefly, viral isolates were used to infect a sub-confluent monolayer of Vero cells at a concentration of 75 plaque forming units (PFU) per well. After 1 hour incubation at 37°C cells were overlaid with CMC medium (4% Carboxymethyl cellulose in PBS) containing a serial dilution of the antiviral drugs acyclovir (ACV), pencyclovir (PCV), cidofovir (CDV) and foscarnet (FOS) or CMC alone, as a no drug control, and incubated for a further 72 hours until plaques became apparent. Cells were fixed with 10% formalin and stained with crystal violet before enumeration of the plaques. The data was then used to determine IC_50_ values for all four drugs using linear regression. Definitions of phenotypic drug susceptibility classification as sensitive or resistant were as follows: ACV, <3μM or >40μM; PCV, <10μM or >40μM; CDV, <24μM or >30μM; FOS, <250μM or >400μM, respectively. Any IC_50_ values falling in between these sensitive and resistant cut-offs were reported as intermediate resistance.

The samples were anonymized by removal of any patient identifiable information and assignment of a non-specific project number before being subjected to genetic characterization as described below.

### Preparation of viral DNA for sequencing

To prepare viral DNA for sequencing we infected confluent monolayers of Vero cells at 5 PFUs/cell for 24 to 48 hours, until cytopathic effect became apparent.

Viral supernatants were harvested by freeze-thawing and passed through a 0.45 μM filter. Four ml of the virus supernatant was incubated with 20 U/ml DNase (Promega) for 3 hours at 37°C before loading onto a 1.5 ml 20% sucrose cushion and centrifuged at 100,000g for 1 hour. Viral pellets were re-suspended in TE containing DNase chelator and extracted with the PureLink® Viral RNA/DNA Mini Kit (Invitrogen) following manufacturer’s instructions.

### 454 and MinION sequencing

Approximately 500 ng of extracted viral nucleic acid from each sample was processed for sequencing by the Roche 454 GS Junior NGS platform following the manufacturer’s protocol for sequencing viral genomic DNA (gDNA). Briefly, the viral gDNA was subjected to fragmentation by nebulization to generate fragments between 500 to 800 base pairs long. The fragmented viral DNA was then used to create a library using the Rapid Library Preparation Kit (Lib-L), which ligates short DNA adaptors. The short DNA adaptors contain unique multiplex identifiers (MIDs), which allow sequencing of multiple samples in a single run. The DNA was amplified by emulsion PCR before sequencing and all 18 samples were multiplexed in a single run on a Roche 454 GS Junior System.

MinION sequencing libraries were prepared according to MAP003-MinION gDNA Sequencing Kit protocol. Briefly, 1 ug of extracted DNA from 7 -previously 454 sequenced- samples was sheared to approximately 10,000bp with g-Tubes (520079, Covaris, Woburn, Massachusetts, USA). After adding the internal control DNA (phage Lambda DNA), we end-repaired the sheared DNA with NEBNext End-Repair module (New England BioLabs), purified it with Agencourt AMPure XP PCR Purification beads (Beckman Coulter) and dA-tailed it with NEBNext dA-tailing module (New England BioLabs). Finally, we ligated the 3’end T-overhanging hairpin- and Y- formed MinION sequencing adapters to the d-A tailed DNA using NEBNext Blunt/TA Ligase (New England BioLabs). We conditioned and loaded the libraries to the sequencer for a 48hours run, renewing them every 12 hours.

### Bioinformatics

Computational analyses were conducted using open source software packages. The.*ssf* files derived from Roche 454 GS Junior platform, were converted to.*fastq* files using *sff2fastq* utility v0.9.2 (https://github.com/indraniel/sff2fastq). The.*fastq* files were quality-controlled with *fastQC* v.0.10.1 (http://www.bioinformatics.bbsrc.ac.uk/projects/fastqc) and the reads were appropriately trimmed using *fastx_trimmer* from *fastx toolkit* v0.0.13 (http://hannonlab.cshl.edu/fastx_toolkit/index.html), as there was an occasional drop in their quality after approximately the 450^th^ nucleotide. The reads were mapped against the HSV strain 17 reference genome [14, 15] using *bwa* v0.7.12 [52] (default settings) and the resulting alignments were visualized with the *Integrated Genomics Viewer* (IGV) v2.3.60 [53].

MinION basecalling was performed via the Metrichor agent (provided by ONT). We converted the.*fast5* reads to.*fasta* files using the poRe v0.5 package for R programming language [54]. We used “Biostrings” Bioconductor package v2.38 to assess the read-length characteristics of the MinION sequencer for each individual experiment. The alignment of the reads was performed with *LAST v581* [55] setting -T = 1, thus allowing only complete reads to be mapped. We converted the resulting.*maf* alignments to.*sam* using “maf-convert” Python script (https://github.com/arq5x/nanopore-scripts/blob/master/maf-convert.py).

We performed the *de novo* assembly of HHV-1 genomes with *MIRA* [33] using either the filtered 454 reads alone, or in combination with the MinION reads and configuring the assembler as: job = genome,denovo,accurate \ -GE:not = 12 -SK:mmhr = 1 -NW:cdrn = no -AS:nop = 3 urd = yes. The hybrid assemblies were further improved using *LINKS* v1.3 [34] (default settings). The continuity of the hybrid contigs over discontinuous 454 contigs was verified by visual inspection of the mapping assemblies of both 454 and MinION reads, setting as a threshold the existence of at least 2 MinION reads spanning the discontinuous 454 contigs for at least 500bp on each side (Fig 5). Unaligned contigs were removed from the output pool and all assemblies were evaluated with QUAST v2.3 [37]. To identify rearrangement events we filtered the QUAST-misaligned contigs with BLAST to exclude those suggesting rearrangements at the beginning or at the end of repetitive regions and the remaining were evaluated by visual inspection with MAUVE v2.3.1 [36].

We used *abacas* v1.3.1 [56] to align the 454 *de novo* generated contigs with the reference genome, using 100Ns to indicate the assembly gaps. We annotated the assemblies using *prokka* v1.11 [57] and we converted the.gbk outputs to.embl formatted flat files using *Artemis* v16.0.0 [58].

We performed the SNP/INDEL and the consensus calling with the *samtools* package v1.2 [38] filtering-in variations with at least 80% read support per position. Using snpEff v3.6c [39] the.vcf files where annotated to the reference genome and non-synonymous SNPS/INDELS were further filtered with snpSift v3.6c [40] to meet the quality and coverage thresholds of >20 and >5, respectively. Using R for basic calculations, we estimated the per-gene mutation frequency by counting the total number of the per-gene variations observed, divided by the length of each protein, using as reference the HSV1 strain 17.

To calculate the mean coverage of 454 reads across the genome, we used *bedtools coverage* v2.24.0 [35]. We calculated the normalised coverage after dividing the per position coverage by the total mapped reads of each.*bam* file. We visualised the coverage plots in comparison to GC content across the genome using DNAplotter v10.2 [59].

## Supporting Information

S1 Fig Nanopore-MinION read-length histograms (log10 transformed) and proportion of total reads mapped to the HHV-1 genome (dark grey). The read-lengths' distribution but also the total mapped reads varied amongst the MinION runs.
(PDF)

S2 Fig Comparative analysis of solo (S) 454-Roche and hybrid (H) 454-MinION assemblies across all the repeat regions of HHV-1 genome (strain17). Regions covered by at least one contig (+) where more in the hybrid assemblies compared to the solo ones, in 3 out of 5 samples tested.
(PDF)
