# Supplementary material for: De Novo Assembly of Human Herpes Virus Type 1 (HHV-1) Genome, Mining of Non-Canonical Structures and Detection of Novel Drug-Resistance Mutations Using Short- and Long-Read Next Generation Sequencing Technologies
Source: PLoS One. 2016 Jun 16;11(6):e0157600. doi: 10.1371/journal.pone.0157600 (PMC4910999; doi:10.1371/journal.pone.0157600)
Supplement: S1 Fig — The read-lengths' distribution but also the total mapped reads varied amongst the MinION runs. (PDF) [file pone.0157600.s001.pdf]

Supplementary Figure 1

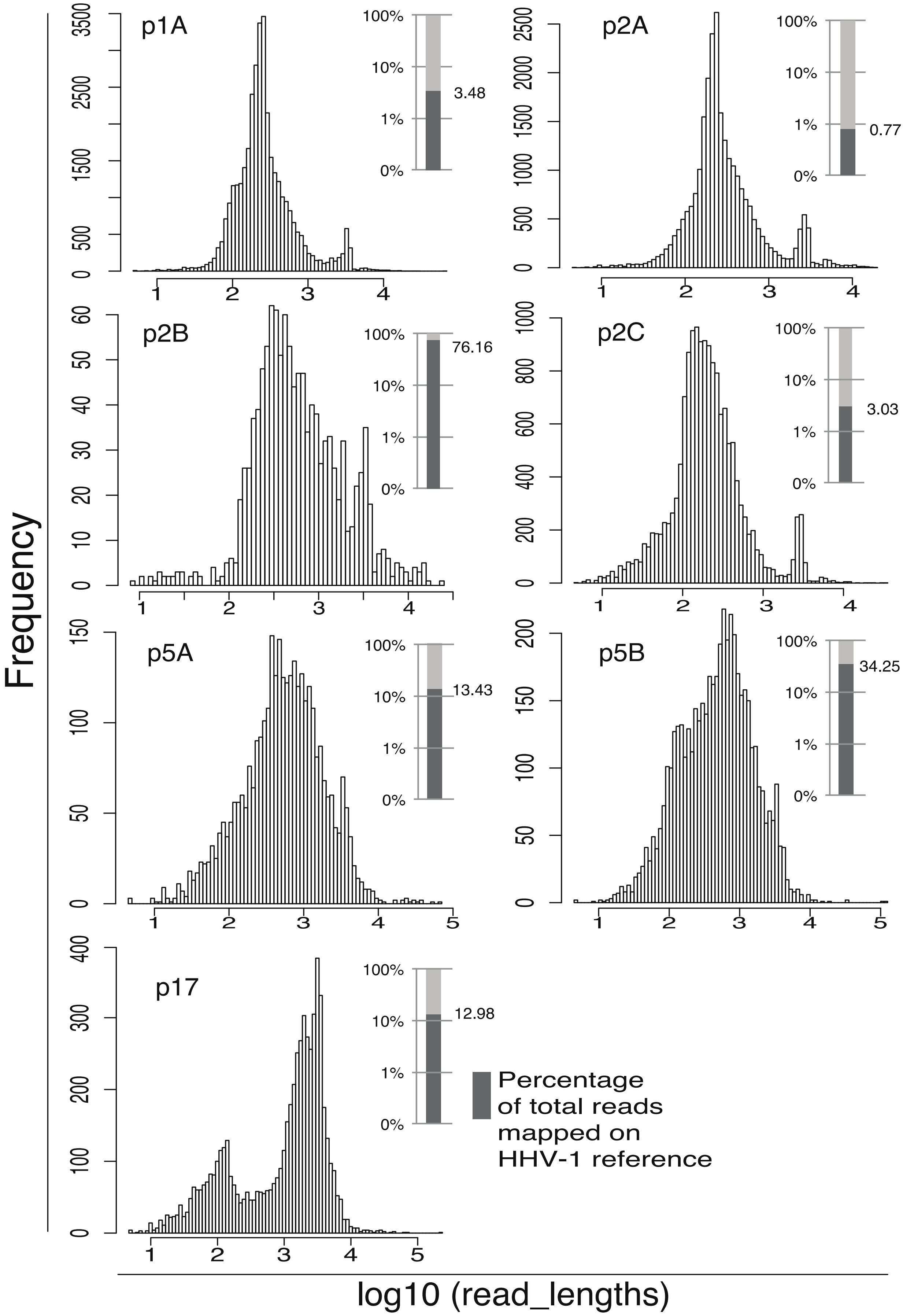

Nanopore-MinION read-length histograms (log10 transformed) and proportion of total reads mapped to the HHV-1 genome (dark grey). The read-lengths' distribution but also the total mapped reads varied amongst the MinION runs.
