# Supplementary material for: De Novo Assembly of Human Herpes Virus Type 1 (HHV-1) Genome, Mining of Non-Canonical Structures and Detection of Novel Drug-Resistance Mutations Using Short- and Long-Read Next Generation Sequencing Technologies
Source: PLoS One. 2016 Jun 16;11(6):e0157600. doi: 10.1371/journal.pone.0157600 (PMC4910999; doi:10.1371/journal.pone.0157600)
Supplement: S2 Fig — Regions covered by at least one contig (+) where more in the hybrid assemblies compared to the solo ones, in 3 out of 5 samples tested. (PDF) [file pone.0157600.s002.pdf]

Supplementary Figure 2

Coordinates		Repeat region	p1A		p2A		p2B		p2C		p5A		p5B		p17	
			S	H	S	H	S	H	S	H	S	H	S	H	S	H
1	397	id2_seq.	-	-	-	-	-	-	-	-	-	-	-	-	-	-
98	320	id3_seq.	-	-	-	-	-	-	-	-	-	-	-	-	-	-
988	1040	id4_TRL reit. set 6	-	-	+	+	+	+	+	+	-	-	-	-	+	+
2465	2623	id10_TRL reit. set 5	+	+	+	+	+	+	+	+	+	+	+	+	+	+
5731	5877	id13_TRL reit. set 4	+	+	+	+	+	+	+	+	+	+	-	-	+	+
8552	8583	id14_TRL reit. set 3	-	-	+	+	+	+	+	+	+	+	+	+	-	-
8806	8826	id15_TRL reit. set 2	-	-	+	+	+	+	+	+	+	+	+	+	+	+
9032	9213	id16_TRL reit. set 1	-	-	-	-	-	-	+	+	-	-	-	-	+	+
62403	62547	id38_oriL palindr_stem_loop	-	-	**-**	**+**	+	+	**-**	**+**	-	-	-	-	-	-
71604	71814	id43_UL reit. set	-	-	+	+	-	-	+	+	**-**	**+**	-	**+**	+	+
117159	117340	id61_IRL reit. set 1	-	-	-	-	-	-	-	-	-	-	-	-	-	-
117547	117567	id63_IRL reit. set 2	-	-	+	+	-	-	-	-	+	+	+	+	-	-
117788	117819	id64_IRL reit. set 3	-	-	+	+	-	-	-	-	+	+	+	+	-	-
120494	120640	id66_IRL reit. set 4	+	+	+	+	+	+	-	-	**+**	**-**	-	-	-	-
123748	123906	id71_IRL reit. set 5	+	+	+	+	+	+	-	-	+	+	+	+	-	-
125331	125383	id74_IRL reit. set 6	-	-	-	-	-	-	-	-	-	-	-	-	-	-
125974	126372	id76_seq.	-	-	-	-	-	-	-	-	-	-	-	-	-	-
126051	126273	id77_seq.	-	-	-	-	-	-	-	-	-	-	-	-	-	-
126571	126709	id78_IRS reit. set 1	-	-	-	-	-	-	-	-	-	-	-	-	-	-
126810	127142	id79_IRS reit. set 2	-	-	-	-	-	-	-	-	-	-	-	-	-	-
132077	132121	id84_oriS palindr_stem_loop	-	-	+	+	+	+	+	+	+	+	+	+	+	+
132388	132513	id88_IRS reit. set 3	-	-	-	-	-	-	-	-	-	-	-	-	+	+
143712	143864	id114_US reit. set 1	-	-	+	+	+	+	+	+	-	-	-	-	+	+
144782	144997	id118_US reit. set 2	+	+	+	+	+	+	+	+	+	+	+	+	+	+
145676	145845	id122_TRS reit. set 3	-	-	-	-	-	-	-	-	-	-	-	-	-	-
146212	146256	id123_oriS palindr_stem_loop	-	-	**-**	**+**	-	-	-	-	-	-	+	+	+	+
151091	151423	id128_TRS reit. set 2	-	-	-	-	-	-	-	-	-	-	-	-	-	-
151524	151662	id129_TRS reit. set 1	-	-	-	-	-	-	-	-	-	-	-	-	-	-
151861	152259	id130_seq.	-	-	-	-	-	-	-	-	-	-	-	-	-	-
151960	152182	id131_seq.	-	-	-	-	-	-	-	-	-	-	-	-	-	-
		**Total loci covered at least ones**	5	5	13	**15**	11	11	10	**11**	10	10	9	**10**	11	11

Comparative analysis of solo (S) 454-Roche and hybrid (H) 454-MinION assemblies across all the repeat regions of HHV-1 genome (strain17). Regions covered by at least one contig (+) where more in the hybrid assemblies compared to the solo ones, in 3 out of 5 samples tested.
